# Karel Maršál 1943–2023

**DOI:** 10.1111/aogs.14564

**Published:** 2023-05-15

**Authors:** Sturla Eik‐Nes, Reynir Tomas Geirsson, Saemundur Gudmundsson, Pentti Jouppila, Torvid Kiserud, Karin Källén, Torben Larsen, Per Olofsson, Per‐Håkan Persson, Ann Thuring, Lil Valentin

**Affiliations:** ^1^ Norway; ^2^ Iceland; ^3^ Sweden; ^4^ Finland; ^5^ Denmark

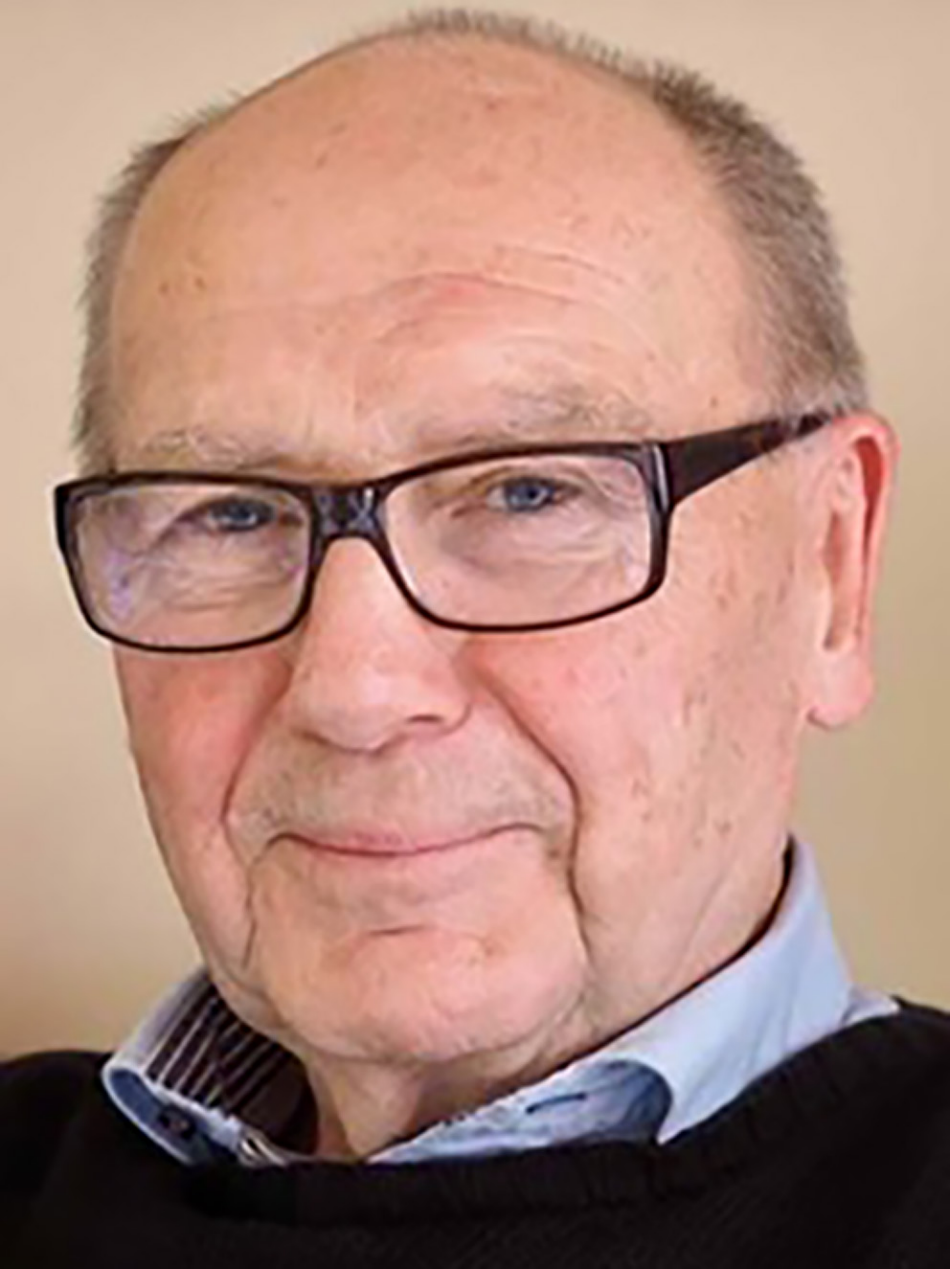

It is with sadness that we received the message that Karel Maršál passed away on March 6, 2023. Karel Maršál, MD, PhD, Professor Emeritus at Lund University and Director of the WHO Collaborating Center in Lund, was born in Prague, former Czechoslovakia, in 1943. After completing his undergraduate studies at Charles University in Prague, he and his fiancée Lida moved as political refugees to Sweden in 1968. They came to Malmö General Hospital in 1973, where Karel gained his specialty in obstetrics and gynecology. Already then, Malmö was a nursery for research on fetal physiology and monitoring, and Karel was soon involved. He defended his PhD thesis, “Ultrasonic Measurements of Fetal Breathing Movements in Man”, at Lund University in 1977.

With the introduction of Doppler ultrasonography to measure blood flow velocity, an era of prolific research commenced, leading to a growing international collaboration under Karel's leadership. In a series of PhD theses, normal fetal circulation was mapped to form the basis for the clinical assessment of the compromised fetus, and for studying the effects of medications and smoking during pregnancy. Experimental work on fetal lambs added further insights into circulatory pathophysiology. The introduction of routine ultrasound in Malmö in 1972 paved the way for identifying and studying fetal growth restriction and its underlying circulatory changes, today a major clinical field. Karel was the Head of the Perinatal Division at the University Hospital in Malmö and his personality as an enthusiastic, persevering, and meticulous scientist earned him a professorship in Malmö in 1991.

In 1997, Karel moved to Lund to become Professor in Obstetrics and Gynecology, and later head of the clinical department. In 1995, he became Honorary Professor at Charles University in Prague, Czech Republic. He also served as Director of the undergraduate program of the Medical School at Lund University and as Chief Medical Officer in the county of Skåne, Sweden. Karel was the main tutor of 21 PhD students and co‐tutor of another 14 research students. He published 354 original papers, 132 book chapters and reviews, and 14 textbooks. He was editor or co‐editor of the *Journal of Maternal and Fetal Investigation*, of *Fetal and Maternal Medicine Review*, and of *Seminars in Fetal and Neonatal Medicine*, and was a member of the editorial board of 10 international scientific journals. His primary areas of experience and research were in perinatology, obstetrics, fetal monitoring, obstetric and gynecologic ultrasound, Doppler ultrasound, fetal physiology, ultrasound safety, and obstetric quality assurance.

Throughout the years, Karel's research was supported by numerous grants, among them from the prestigious Swedish Research Council. He was the principal investigator in numerous national and international multicenter studies, such as the EXPRESS study on extremely preterm infants in Sweden, and he was national coordinator for Sweden in three EU studies. Karel served as president of the International Society of Ultrasound in Obstetrics and Gynecology, he was an honorary member of various national and international institutions and a board member of European Federation of Societies for Ultrasound in Medicine and Biology, World Federation for Ultrasound in Medicine and Biology, and other societies. In the past, he was the main organizer of 15 international scientific congresses, including two world congresses on ultrasound in obstetrics and gynecology. He received several prestigious awards, such as the Ian Donald Gold Medal and the Haackert Gold Medal in Prenatal Medicine. Karel was elected *Doctor Honoris Causae* at universities in Olomouc, Czech Republic, and Poznan, Poland. He was course leader of 38 national and international postgraduate courses in perinatal medicine, obstetrics, ultrasound, and obstetric Doppler ultrasound. His last participation in such an event was in February 2023.

Karel's wife, Lida, gave him unending support throughout his career. Had Karel not injured his leg in a skiing accident, their paths may never have crossed. He had to take a break from medical school, and when he returned to his studies, he and Lida became classmates.

Innumerable mothers and children all over the globe have benefited from Karel's ground‐breaking scientific work, leadership, and deeds. His 50‐year career as a clinician, scientist, teacher, coach, role model, and—most of all—the best friend of the unborn baby is now at an end. Among his friends and co‐workers, he will be vividly remembered as a quietly jovial, sharp‐witted gentleman with a human perspective and a friendly attitude toward each of us. He is sorely missed.

